# Evaluation of Extraction Procedure of PCDD/Fs, PCBs and Chlorobenzenes from Activated Carbon Fibers (ACFs)

**DOI:** 10.3390/molecules26216407

**Published:** 2021-10-23

**Authors:** Marina Cerasa, Ettore Guerriero, Silvia Mosca

**Affiliations:** Institute on Atmospheric Pollution (IIA), National Research Council of Italy (CNR), Via Salaria km 29,300, Monterotondo, 00015 Rome, Italy; marina.cerasa@iia.cnr.it (M.C.); guerriero@iia.cnr.it (E.G.)

**Keywords:** extraction, active carbon fiber, PCDD/Fs, PCBs, CBs, ACF, QA/QC

## Abstract

Active carbon-based sorbents are well known and are used in analytical chemistry. Activated carbon fibers (ACFs) are mainly used as abatement systems in industrial emission pollution control. The objective of this study was to extend the use of ACFs in analytical chemistry for the analysis of polychlorodibenzo-p-dioxins (PCDDs), polychlorodibenzofurans (PCDFs), dioxin-like polychlorobiphenyls (PCBs), and chlorobenzenes (CBs). For this purpose, the extraction efficiency was evaluated based on the QA/QC criteria defined by EPA/ISO reference methods on ^13^C-standards recovery rates. The procedures tested were ultrasonic assisted extraction (UAE), Soxhlet extraction (SE), accelerated solvent extraction (ASE), and microwave-assisted extraction (MAE). Each experiment was performed in triplicate to ensure the repeatability of the results, and a second extraction assessed the complete extraction. The comparison of the results of each set of experiments with the minimum requirements of the reference methods for each class of compounds led to SE being chosen as the best technique. SE with toluene resulted in a reduction of time and costs and with respect to the other investigated techniques. The present work demonstrated that ACFs can be used in environmental fields means of both prevention and control (exploiting the adsorbent characteristics) and for analytical purposes (exploiting the desorption) for the described chlorinated classes of pollutants.

## 1. Introduction

Activated carbon fibers (ACFs) are a set of promising microporous materials and are one amongst the most effective adsorbents in adsorption applications [1]. Their high adsorption capacities are due to their nanostructure, high homogeneous porosity, high specific surface area (SSA), and narrow pore size distribution [2]. Moreover, the material is lightweight (low density per m^2^), it can be wrapped in different textiles (easy to handle), and it is fireproof [3]. All of these characteristics enable ACFs to be widely used in various fields such as pollutant removal and air conditioning filters, vapour sensors, biomedical applications, capacitors, carbon molecular sieves, and electrodes [4,5,6,7,8,9,10].

Both the high SSA and the evenly distributed porosity on the surface make ACF a suitable candidate for reversible mass transfer (adsorption/desorption), especially when considering the possibility of desorbing identical contaminants that have been previously adsorbed. This can be an advantageous property that can be used to extract the compounds trapped within the ACF more easily, resulting in better recoveries [11,12]. Since the literature data are mostly based on the use of ACFs for environmental remediation and bioenergy applications and not for analytical purposes [13,14,15], Cerasa et al. carried out specific tests to verify the technical files of vendors and studied their chemical and physical characteristics for analytical use [16].

The reference air sampling methods [17,18,19] involve the use of a quartz fiber filter (QFF) and polyurethane foam (PUF) in series to trap the fraction of the pollutants in the particulate and in the gaseous phase, respectively.

Based on these considerations, the objective of this work was to establish efficient extraction procedures for polychlorodibenzo-p-dioxins (PCDDs), polychlorodibenzofurans (PCDFs), dioxin-like polychlorobiphenyls (PCBs), pentachlorobenzene (PeCB) and hexachlorobenzene (HCB) using the most common solvent extraction techniques to compare the obtained yields and to describe the processes. Considering the requirements of the standard methods taken as a reference (EPA TO-4A, EPA TO-9A and iso 16000-13), ensuring the extraction of the aforementioned classes of compounds represents the first step that needs to be taken before ACF can be considered as a sampling adsorbent and before it can be compared with a reference.

## 2. Materials and Methods

### 2.1. Standards and Reagents

High purity solvents used in extraction and clean-up (toluene, methanol, acetone, *n*-hexane (*n*-HEX), and dichloromethane (DCM)) were purchased from a local distributor, ROMIL Ltd. (Cambridge, GB).

Stock solutions of ^13^C-labelled compounds were purchased from a local distributor of Wellington Labs (Guelph, Canada) and were used in isotopic dilution analysis as follows:

Extraction Standard Solution (ES Solution) containing PCDD/PCDF (EN 1948 ES), PCBs (WP-LCS), HCB and PeCB (MCBS).

Injection Standard Solution (IS Solution) containing PCDD/Fs (EN 1948 IS) and PCBs (WP-ISS).

Ready-to-use calibration kit for PCDD/Fs and PCBs.

The clean-up was conducted using microcolumns packed with MP EcoChrom^TM^ Alumina B—Super I for Dioxin Analysis purchased from a local distributor of MP Biomedical LLC (Santa Ana, CA, USA).

### 2.2. Active Carbon-Based Material

The physical-chemical characterization of the active carbon-based material (Kynol^®^ novoloid fibers, Kynol Europa GmbH, Hamburg, Germany) used in this work was described by Cerasa et al. [16]. Briefly, the material (ACF) is based on cured phenol-aldehyde fibers that have a specific surface area of about 2500 m^2^/g and a microporosity that is uniformly distributed on the surface. The chemical-physical characteristics reported here are strictly related to the extraction tests. The adsorbing capacity of the activated carbon fibers (ACF) and the reversibility of the process are mainly related to its porosity (size, uniformity, and distribution), to the specific surface area, and to the active chemical groups.

The ACF filters were cut to the same dimensions as the quartz fiber filter (QFF) used on a high-volume sampling head, i.e., 102 mm diameter (1.564 ± 0.002 g). The filters were pre-cleansed in Soxhlet with toluene for 24 h and were left to dry overnight at 150 °C under N_2_ flow.

### 2.3. Extraction Procedures

The extraction procedures tested in this paper were (a) ultrasonic assisted extraction (UAE), (b) Soxhlet extraction (SE), (c) accelerated solvent extraction (ASE) and (d) microwave-assisted extraction (MAE).

In all the cases, the ACF filters were spiked with the ES Solution, and the extraction was performed after 10 min. Each experiment was performed in triplicate, and the data were subjected to calculations of the mean ± standard deviation. The mean values were used to create the graphs.

A second extraction was repeated once more on the same filter to assess the complete extraction. According with ISO method “the extract of a repeated extraction procedure shall not contain more than 5% of the amount of any individual native congener compared with the first extraction” [20]. This test was fundamental to ensure the capability of this material to be used for analytical aims. 

#### 2.3.1. Ultrasonic Assisted Extraction (UAE)

Two pre-spiked ACF filters were placed in separate volumetric flasks with 50 mL of toluene and both were immersed in an ultrasonic bath at 40 °C. One was sonicated for 5 min, and the other one was sonicated for 10 min. The two supernatants were filtered with a paper filter and were collected in two different vials; the whole procedure was repeated two more times, with each fraction being collected separately. 

#### 2.3.2. Soxhlet Extraction (SE)

A pre-spiked ACF filter was placed in a paper thimble and was extracted using Soxhlet with toluene for 36 h. Given the hygroscopicity of the ACF, tests with the mixture of 10% (*v*/*v*) methanol or acetone in toluene were included. Since there were no improvements in the results attributable to this test, the data are irrelevant for the purposes of this research and will be omitted.

#### 2.3.3. Accelerated Solvent Extraction (ASE)

The extractions were performed with toluene in 11 mL stainless-steel cells at 100 atm pressure by three static cycles, which were conducted at 200 °C for 5 min. After the static time, toluene was flushed through the cell to remove the extracted analytes. The amount of solvent used for the flush was about 70% of the volume of the cell used for the extraction, and a third of the total flush volume was pumped through the cell at the conclusion of each static cycle. The extracted analytes were purged from the sample cell using pressurized nitrogen for 100 s and were collected in a 40 mL vial.

#### 2.3.4. Microwaves Assisted Exaction (MAE)

The extraction was conducted with a microwave (ETHOS 1-Microwave Digestion/Extraction Labstation, Milestone Srl, Bergamo, Italy) in toluene. The program temperature was set as follows: initial temperature and hold 19 °C for 0 s; initial ramp to 120 °C at 20 °C per minute (800 W); second hold 120 °C for 20 min (800 W); 10 min of ventilation (0 W).

### 2.4. Clean-Up

The extracts were concentrated with a rotary evaporator and were then placed under a gentle N_2_ flow in a thermostatic bath that was between 45 ± 5 °C. The clean-up consisted of an alumina microcolumn to separate the PCDD/Fs from dl-PCB and CBs.

A Pasteur microcolumn that had been manually packed with alumina was washed with 10 mL *n*-HEX:DCM (50:50 *v*/*v*) and conditioned with 10 mL *n*-HEX.

The sample was loaded at the head of the column and 20 mL *n*-HEX (not collected) washed the nonpolar interferents. CBs and PCBs were eluted with 20 mL *n*-Hex:DCM (94:6 *v*/*v*) and PCDD/PCDFs with 20 mL di *n*-HEX:DCM (50:50 *v*/*v*).

The collected fractions were concentrated under N_2_ flow in a thermostatic bath at 45 ± 5 °C, and IS Solution was added prior to injection into the GC-MS/MS.

### 2.5. Quality Assurance/Quality Control (QA/QC)

Before the samples were analyzed, a calibration curve was injected with five stock solutions that contained all of the native and ^13^C-labelled PCDD/PCDFs, PCBs, and CBs in triplicate. It was used to calculate the relative response factors of the analytes (*rrf_i_*) with respect to the corresponding labeled compounds. The RRFs of the CBs were calculated in reference to ^13^C-PeCB and ^13^C-HCB.

The extraction efficiency of the tested methods was assessed through the comparison of the recovery rates (R%) of the compounds contained in the ES Solution, as defined by the reference (Table 1).

## 3. Results

To evaluate the different extraction methods, the applicability, reliability, reproducibility, and overall efficiency were compared. The applicability and the reliability of the methods were assessed based on the compliance with the minimum requirements provided by standard reference ISO and EPA methods. The reproducibility was assessed on the overall average reproducibility of each method and within each compound. The overall efficiency was assessed by considering the time, the cost, and the environmental impact of each method.

**Figure 1:**
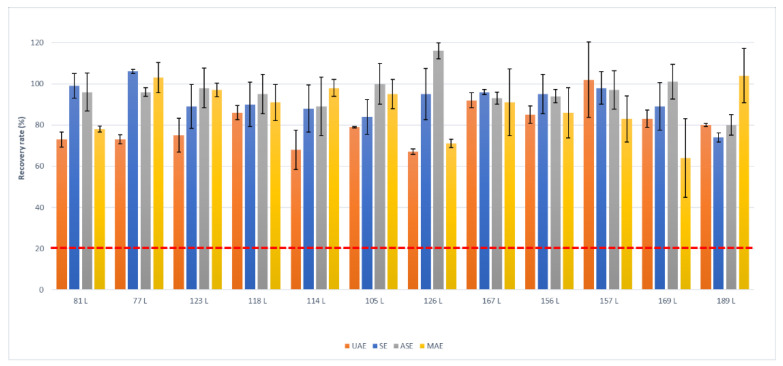
Recovery rates (%) and standard deviation of ^13^C-dl-PCBs (labeled with “L”) by ultrasonic assisted (UAE), Soxhlet (SE), accelerated solvent (ASE), and Microwave assisted (MAE) extractions. Each procedure was performed in triplicate. The dashed red line is the minimum acceptable recovery rate (ISO16000-14).

**Figure 2:**
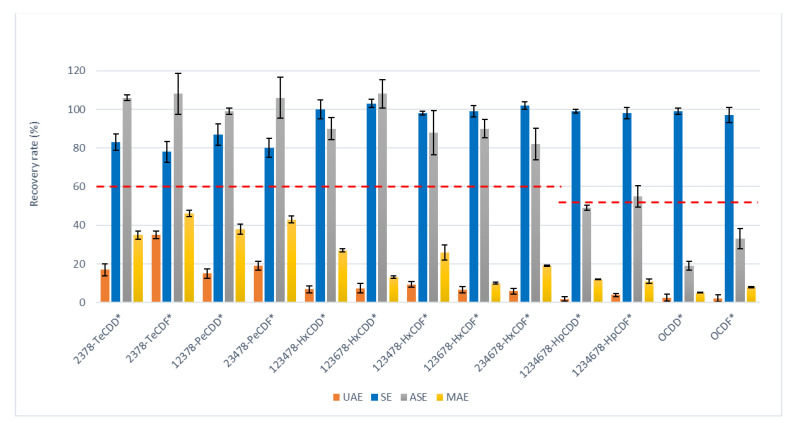
Recovery rates (%) and standard deviation of ^13^C-PCDD/Fs (labeled with “*”) by ultrasonic assisted (UAE), Soxhlet (SE), accelerated solvent (ASE), and Microwave assisted (MAE) extractions. Each procedure was performed in triplicate. The dashed red line is the minimum acceptable recovery rate (ISO16000-14).

**Figure 3:**
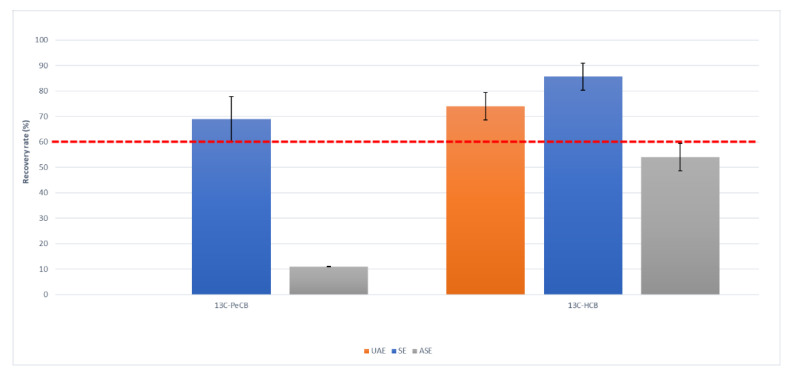
Recovery rates (%) and standard deviation of ^13^C-PeCB and ^13^C-HCB by ultrasonic assisted (UAE), Soxhlet (SE) and accelerated solvent (ASE) extraction. Each procedure was performed in triplicate.

Figure 1, Figure 2 and Figure 3 show recovery rates and the relative standard deviation on triplicate analysis, and the minimum requirements of reference methods (red dashed line) for each class of compounds that were studied.

For PCBs, the extraction techniques yielded comparable results, and the overall average extraction recovery rate was 90%, as observed in Figure 1. On the other hand, the results for PCDD/Fs were generally low, with the exception of SE, which complied with the minimum requirements (Figure 2). Soxhlet is also the extraction method that has been confirmed for chlorobenzenes (Figure 3), as it is the only method that quantitatively extracts PeCB. No recoveries were obtained from the microwave technique since the high temperatures that were reached could have become degraded or lost due to the volatilization of the analytes.

Regarding PCDD/Fs, MAE performed worse than ASE. Although the method is quite reproducible for PCDD/Fs (low SD and RSD%), the R%s do not meet the requirements imposed by the reference methods. Furthermore, the average R%s vary according to the congener, as shown by the SD of the total recovery to be equal to 30%. If the higher recoveries obtained with the extraction in Soxhlet compared to the ASE for PCDD/Fs justified the preference of the former, the advantage is even greater when compared to microwave: the Soxhlet extracted about 63% more PCDD/Fs than the microwave technique did, with a variability on the total Rs of PCDD/Fs of only 8%.

The RSD values of PCBs for SE, ASE, and MAE were about 10%; the values of the PCDD/Fs for SE were about half those of ASE and MAE. It can therefore be argued that SE is the technique among those tested that produces better or the most comparable recoveries and provides a lower relative standard deviation (index of greater precision of the technique).

## 4. Discussion

**Figure 4:**
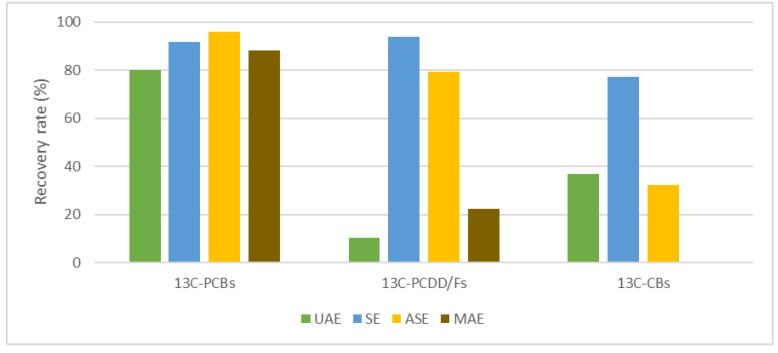
Average overall recovery rates of ^13^C-PCDD/Fs, ^13^C-dlPCBs and ^13^C-CBs by ultrasonic assisted (UAE), Soxhlet (SE), accelerated solvent (ASE) and Microwave assisted (MAE) extraction.

Comparing the average percentage recoveries for each class of contaminants (Figure 4), it can be observed that the Soxhlet extraction technique (SE) is the best method among those that were tested.

A phenomenon that was observed during MAE was the high temperature in point-like areas, specifically at the borders of the ACF membranes to be extracted, which produced distortions in the microwave Teflon (TFM) vessels. The interaction between microwaves and ACF, which is an electrical conductor, sees the free delocalized electrons moving freely on the surface of the material, and this flow can heat the material on its own (Maxwell–Wagner effect) [21,22,23,24]. Despite the ACF being immersed in toluene (boiling point 110 °C), the Teflon vessels presented deformation and fissures at the end of the extraction cycle. This means that locally, the temperature could have reached values higher than those of the maximum Teflon temperature (260°C, according to the manufacturer’s specifications). Although the extractions were set up in the same way, the results were not comparable. The boiling reached by the solution due to the rise in temperatures raised the ACF disks, causing them to come out of the solution. This did not allow (1) the transfer of thermal energy to the extraction solvent or (2) a comparable extraction of the homogeneous ES Solution.

In general, when extracting these contaminants from solid samples (i.e., soils, foods, etc.) ASE and MAE yield higher results compared to SE, and the highest recovery rates are determined for the more Cl-substituted congeners, which have large octanol-water partitioning coefficients (K_ow_) [25,26]. In this work, this is not true, and this phenomenon could depend on the interactions between the contaminants and the matrix, i.e., ACF.

The chemical surface characterization of ACF caused by Boehm titration showed a strong acidic component that was specifically linked to the carboxyl groups and had a strong basic component due to the pyrone groups, whose oxygens confer a negative charge to the material [9,13,14,27,28,29,30].

Guo et al. (2016) supposed that the adsorption reaction between chlorinated hydrocarbons and the carbon surface is due to the lactone groups. PCDD/Fs and PCBs are polychlorinated compounds, and ACF has few lactone groups [31]. It is reasonable to assume that desorption of these compounds is related to the proportion between the acid and the basic component. It is also interesting to underline that the acidic groups are related to the adsorption ratio, which is directly proportional to pH values [32].

The extent of adsorption/desorption is also dependent on the molecular diameter of the analyte and the porous diameter of the adsorbent.

According to literature, when considering large organic molecules such as PCDD/Fs and PCBs, the reversibility of the adsorption on ACF is possible because the pore width (about 1.2 nm) is narrower than the adsorbed molecules. Furthermore, pore shape does not allow these planar molecules to be trapped in the micropores of the material [33]. Although the structure of PCBs differs according to the number of chlorine substitutions, the molecular size is approximately 1.4 nm along the major axis and 0.8 nm along the minor axis and is 0.4 and 0.8 nm thick. The study by Kawashima et al. demonstrated that materials with an SSA of 700 and 1200 m^2^/g and a pore diameter of approximately 0.7 and 0.8 nm are suitable for the adsorption of PCBs [32]. According to Li et al., however, the pore diameter of the adsorbent should be of the mesopore order (2 and 5 nm) in order to completely remove PCDD/Fs (whose diameter is approximately 0.35 and 1.37 nm) and PCBs [34]. In addition to the porous diameter, its distribution and pore type is the second aspect to be evaluated for molecule desorption. Therefore, if the porosity is deeply branched and if the pores have different sizes, the compounds can penetrate and can be trapped in the larger pores. This is the reason why ACF has good reversible adsorption on both PCDD/Fs and PCBs. The pores have a diameter of 1.3 nm and are evenly distributed on the surface, which does not allow these compounds to penetrate deeply, favouring surface adsorption [16]. Moreover, the texture greatly influences the contact points so that the adsorption sites can increase according to the armour. Adsorption/desorption are phenomena that depend not only on physical but also on chemical interactions. Unsaturated carbon atoms with unpaired electrons characterize the basal planes of the ACF surface. Oxygen-containing heteroatoms are usually bonded to these electrons. Aromatic compounds are adsorbed on activated carbon surfaces through π-π dispersion interactions with graphene layers [15]. The functional oxygen groups at the edges of these layers provide sites for the adsorption of hydrophilic species and can affect the adsorption of the hydrophobic compounds on the graphene layers. This is exactly what happens to PCDD/Fs and PCBs: the carboxyl groups tend to attract the p-electrons of the graphene basal plane, which, by reducing the donation of the π-π electrons, reduce the adsorbate–adsorbent interaction strength. The lower the SSA, the more the π-π interactions (involved in electron donation from carbon to aromatic adsorbate) are weakened by the functional groups at the head of the graphene layers. The enthalpy of the adsorption augments higher surface coverage because it influences the π-π adsorbate–adsorbate interactions. As it is well known, chlorine is characterized both by a weak resonance effect (which makes the electronic pair of the halogen able to interact with the electronic system of the benzene ring) and by a more important and prevalent inductive effect, which depletes the aromatic system. The nature of the predominant electron withdrawing group (EWG) of the chlorine makes the interaction between the graphene layers and the PCDD/Fs and PCBs weaker, justifying their greater desorption [35].

## 5. Conclusions

ACF is a material that is commonly used as an adsorbent/remover for pollutants. In this work, it was demonstrated that it is possible to desorb PCDD/Fs, PCBs, and chlorobenzenes and to extend its use for analytical purposes (i.e., air sampling). A comparison of the most common extraction procedures for chlorinated organic pollutants was presented in this study and considered the minimum requirements of standard reference methods for air sampling. The data presented indicated that by using Soxhlet extraction with toluene, we can achieve the best results among the tested methods in terms of recoveries for each class of contaminants.

## 6. Patents

Filing of an international patent application n. PCT/IB2021/056894 of 29 July 2021, with the claim of the priority of the Italian application n. 102020000019936 filed on 11 August 2020.

## Figures and Tables

**Table 1:** Method precision is determined by %RSD for each tested technique and is based on triplicate tests. The criterion of acceptance is <15%.

Compounds	Recovery Rates (%)	Reference Method
^13^C-dl-PCB	20–150	ISO 16000-14 [20]
^13^C-TeCDD/Fs, ^13^C-PeCDD/Fs, ^13^C-Hx CDD/Fs	50–130	
^13^C-HpCDD/Fs, ^13^C-OCDD/Fs	40–130	
^13^C-PeCB, ^13^C-HCB	60–120	EPA TO4A [17]

## Sample Availability

Samples of the compounds are not available from the authors.
